# Ablation of Outflow Tract Arrhythmias in Patients With and Without Structural Heart Disease—A Comparative Analysis

**DOI:** 10.3389/fcvm.2022.910042

**Published:** 2022-05-25

**Authors:** Ruben Schleberger, Jan Riess, Anika Brauer, Hans O. Pinnschmidt, Laura Rottner, Fabian Moser, Julia Moser, Shinwan Kany, Ilaria My, Marc D. Lemoine, Bruno Reissmann, Christian Meyer, Andreas Metzner, Feifan Ouyang, Paulus Kirchhof, Andreas Rillig

**Affiliations:** ^1^Department of Cardiology, University Heart and Vascular Center Hamburg, University Medical Center Hamburg-Eppendorf, Hamburg, Germany; ^2^DZHK (German Center for Cardiovascular Research), Partner Site Hamburg/Kiel/Lübeck, Berlin, Germany; ^3^Institute of Medical Biometry and Epidemiology, Center of Experimental Medicine, University Medical Center Hamburg-Eppendorf, Hamburg, Germany; ^4^Department of Cardiology, Cardiac Neuro- and Electrophysiology Research Consortium, Protestant Hospital Düsseldorf, Düsseldorf, Germany; ^5^Cardiac Neuro- and Electrophysiology Research Consortium, Medical Faculty, Heinrich Heine University Düsseldorf, Düsseldorf, Germany; ^6^Hong Kong Asian Medical Group, Hong Kong, China; ^7^Institute of Cardiovascular Sciences, University of Birmingham, Birmingham, United Kingdom

**Keywords:** catheter ablation, outflow tract arrhythmia, procedural outcome, structural heart disease, ventricular tachycardia, premature ventricular complexes

## Abstract

**Introduction:**

Catheter ablation of ventricular arrhythmias emerging from the ventricular outflow tracts and adjacent structures is very effective and considered almost curative in patients without structural heart disease (SHD). Outcomes of patients with SHD undergoing ablation of outflow tract arrhythmias are not known.

**Methods:**

Consecutive patients (2019–2021) undergoing catheter ablation of ventricular arrhythmias in a single high-volume center were retrospectively analyzed. Patients with ablation of outflow tract arrhythmias were identified and divided in individuals with and without SHD. Procedural parameters and acute outcome were compared.

**Results:**

We identified 215 patients with outflow tract arrhythmias (35.3% female, mean age 58.3 ± 16.0 years). Of those, 93 (43.3%) had SHD. Patients with SHD and outflow tract arrhythmias were older (65.0 ± 12.8 vs. 53.3 ± 16.3 years; *p* < 0.001), more often male (82.8 vs. 50.0%; *p* < 0.001) and had more comorbidities than patients without SHD (arterial hypertension: 62.4 vs. 34.4%, *p* < 0.001; diabetes: 22.6 vs. 8.2%, *p* = 0.005; chronic lung disease: 20.4 vs. 7.4%, *p* = 0.007). Outflow tract arrhythmias in patients with SHD had their origin more often in the left ventricle (68.8 vs. 53.3%, *p* = 0.025). The acute success rate was similar in both patient groups (93.4 vs. 94.2%, *p* = 0.781). Patients with SHD were discharged later {median length of hospital stay with SHD 5 [6 (interquartile range)] days, without SHD 2 [4] days, *p* < 0.001}. Periprocedural complications were numerically more frequent in patients with SHD [with SHD 12 (12.9%), without SHD 8 (6.6%), *p* = 0.154].

**Conclusion:**

Outflow tract arrhythmia ablation has a high success rate irrespective of the presence of SHD. Longer hospital stay and potentially a higher risk of periprocedural complications should be considered when discussing this treatment option with patients.

## Introduction

Despite advances in mapping and ablation technology, catheter ablation of ventricular arrhythmias remains difficult (1). Acute and long-term outcome vary depending on patient selection (2–5). Large substrates and multiple origins of ventricular tachycardias (VT) are reasons for a low success rate, especially in patients with structural heart disease (SHD) (6). Subtypes with excellent outcome are ventricular arrhythmias emerging from the right ventricular outflow tract and -to a lesser extent- also from the left ventricular outflow tract including its adjacent structures like aortic cusps, aortomitral continuity and left ventricular summit (7–10). Many patients present with such outflow tract arrhythmias and SHD. These arrhythmias are considered more difficult to treat by ablation due to presence of myocardial substrate areas and might differ from idiopathic outflow tract arrhythmias because of a reentry rather than focal mechanism (11). Whether the outcome of outflow tract arrhythmias in patients with SHD is comparable to that in patients without SHD is not known. We therefore analyzed procedural outcomes in a large series of patients undergoing ablation of outflow tract arrhythmias with and without SHD.

## Methods

### Study Design

**Figure 1:**
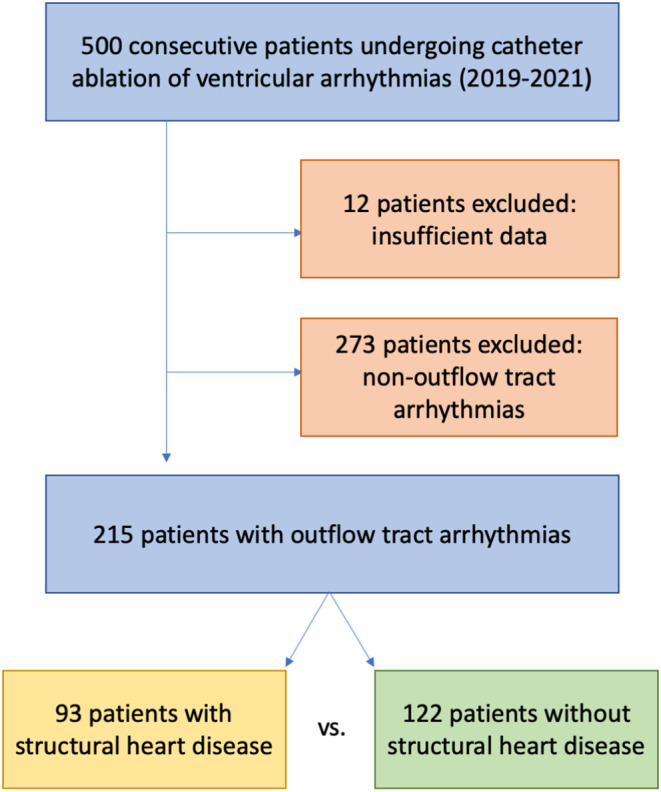
Flow chart of the analyzed patient cohort.

Consecutive patients presenting for ablation of ventricular arrhythmias at our tertiary care center between January 2019 and October 2021 were retrospectively analyzed. Patients with insufficient available procedural data were excluded from analysis (see Figure 1). Data collection and analysis were approved by the ethics committee of the Medical Association Hamburg. All patients gave written informed consent to data analysis and procedure.

Patients with outflow tract arrhythmias were identified and classified as patients with or without SHD. All patients had cardiac imaging (e.g., echocardiography, cardiac magnetic resonance imaging or computer tomography before the procedure). SHD was defined as history of or present evidence of abnormal systolic or diastolic ventricular function or more than mild abnormality of cardiac dimensions and diameters, abnormalities of myocardial texture, known coronary artery disease, higher than mild valve regurgitation or any valve stenosis or congenital heart disease. Baseline parameters, procedural characteristics and acute outcome were analyzed.

### Electrophysiological Evaluation and Instrumentation

The procedures were performed under conscious sedation or general anesthesia. Reversible causes of arrhythmia were excluded before the procedure. Detailed procedural methods have been described before (12). Electroanatomical three-dimensional mapping was performed using the Carto mapping system (Carto^®^ 3 System, Biosense Webster, Irvine, CA, USA). Unfractionated heparin was administered intravenously to maintain an activated clotting time >300 s in patients with left ventricular access, all others received a heparin bolus of 3.000 IE in the beginning of the procedure. At first an electroanatomical voltage map was acquired with a steerable single-tip ablation catheter (THERMOCOOL^®^, D- or F-Type, 2-5-2 mm spacing, Biosense Webster) or a multipolar mapping catheter (PENTARAY™ NAV eco Catheter, Biosense Webster) through retrograde (aortic) and/or antegrade (transseptal) access (13). Local abnormal ventricular activity, including fractionated and late potentials as well as areas of scar, were marked whenever appropriate and later considered for ablation. In line with previous studies for scar demarcation a bipolar endocardial voltage of 0.5/1.5 mV with individual adaptation was chosen (12, 14). All voltage maps were generated during sinus rhythm or paced rhythm from coronary sinus or right ventricle. In case of presumed VT a 6 French quadripolar diagnostic catheter, placed in the right ventricular apex was used to induce VT by programmed stimulation with a fixed stimulation protocol consisting of three basic cycle lengths (510, 440, 370 ms) and up to three added extra stimuli (lowest coupling interval 200 ms). In patients presenting with VT at the beginning of the procedure, activation/entrainment mapping was conducted before substrate mapping. Pace mapping and activation mapping was performed at the operator's discretion, especially in patients presenting for ablation of premature ventricular complexes (PVC). In case of absence of PVC or non-inducibility of VT, sedation was reduced and/or orciprenaline was administered continuously until a 20% increase of the baseline heartrate was achieved. Then stimulation was repeated. Radiofrequency current was used for complete abolition of all abnormal electrograms including fractionated potentials, highly fractionated potentials, late potentials, and fractionated late potentials in patients with abnormal myocardial substrate (15, 16). Radiofrequency current was applied with a maximum power of 45 Watts (upper temperature limit was set to 48°C) at an irrigation rate of 17–30 mL per min. If the ablation site was in possible proximity to the coronary arteries, coronary angiography was performed for visualization before ablation. Subsequently, targeted regions were remapped to demonstrate elimination of the respective electrograms. We aimed to achieve the combined procedural endpoint of VT non-inducibility, freedom from spontaneous PVC and substrate modification. In patients that required initial orciprenaline infusion due to absence of spontaneous arrhythmia, infusion was repeated at the end of procedure to confirm ablation success. At the end of the procedure, all sheaths were removed, and the puncture site was compressed manually until complete haemostasis was achieved. Consequently, a compression bandage was applied. An Angio-Seal™ device (Terumo Interventional Systems, Terumo Europe, Löwen, Belgium) was used at the operator's discretion according to the manufacturer's instructions.

All patients were connected to telemetry monitoring on a specialized heart rhythm ward or intensive/intermediate care ward for at least 18 h after the procedure. The monitor records were reviewed by a physician before discharge and PVC, or VT episodes were analyzed and quantified. In case of suspected recurrence, Holter ECG was performed.

After discharge, amiodarone and other antiarrhythmic drugs were continued at the operator's discretion. A recurrence was defined as any sustained VT or recurrence of the targeted PVC documented by device interrogation or ECG.

The electronic patient charts were reviewed by a senior electrophysiologist for the acquisition of patient characteristics, procedural parameters, and acute outcome.

### Statistical Analysis

Descriptive statistics are presented as count and percentage for categorical variables and as mean ± standard deviation or median [interquartile range (IQR)] for continuous variables. The distribution of numeric values was assessed visually using histograms. The baseline and treatment characteristics of patients with and without SHD were compared with Fisher's exact test or Mann-Whitney *U*-test, as appropriate. Cox proportional hazards models were used to assess the relationships between covariates and length of stay or procedure duration. Uni- as well as multivariable Cox-regression analyses were performed. In the multivariable analyses, the grouping variable “structural heart disease” was forced into the model equations while all other covariates were selected following the forward-stepwise variable selection method. Hazard ratios with 95% confidence intervals as well as Wald *P*-values are presented in forest plots. The reported *p-*values are used as descriptive measures only. Significance level α was set to 5%. Statistical analyses were performed using SPSS v. 28.0.1.0 (IBM corporation, USA).

## Results

### Total Study Population

A total of 500 patients were identified. Twelve had insufficient data (Figure 1). The patients were male in 71.7% (350/488) and had a mean age of 61.1 ± 15 years. Baseline characteristics of the total cohort are presented in Supplementary Table 1.

**Table 1:** Baseline characteristics of patients with outflow tract arrhythmias.

**Variable**	**All** **(*n* = 215)**	**With structural heart disease** **(*n* = 93)**	**Without structural heart disease** **(*n* = 122)**	***p*** **-value**
Age (years)	58.3 ± 16	65.0 ± 12.8	53.3 ± 16.3	<0.001
Body-mass-index (kg/m^2^)	27.3 ± 5.5	28.6 ± 6.1	26.2 ± 4.8	0.001
Gender (male)	64.2%	82.8%	50.0%	<0.001
**Admission type**				0.036
Elective	75.8%	68.8%	81.1%	
Urgent	24.2%	31.2%	18.8%	
**Admission ward**				0.133
Normal ward	93.5%	90.3%	95.9%	
Intermediate care	2.8%	5.4%	0.8%	
Intensive care	3.7%	4.3%	3.3%	
**Critical events before admission**
Electrical storm	16.7%	16.7%	16.7%	1.000
Acute heart failure	10.7%	17.2%	5.7%	0.013
**Structural heart disease**	43.3%	100.0%	0.0%	
Ischemic		43.0%		
Non-ischemic		57.0%		
LV-EF (%)	50 ± 14	40 ± 14	57 ± 8	<0.001
TAPSE (mm)	21.1 ± 5.5	20.7 ± 5.9	23.3 ± 4.8	0.001
**ICD**	20.0%	41.9%	3.3%	<0.001
CRT-D	9.8%	21.5%	0.8%	
Pacemaker	1.9%	2.2%	1.6%	1.000
**Comorbidities**
Arterial hypertension	46.5%	62.4%	34.4%	<0.001
Diabetes mellitus	14.4%	22.6%	8.2%	0.005
Chronic kidney disease	26.0%	41.9%	13.9%	<0.001
Chronic lung disease	13.0%	20.4%	7.4%	0.007
Chronic liver disease	4.2%	6.5%	2.5%	0.180
Atrial Fibrillation	20.9%	31.2%	13.1%	0.002
CHA_2_DS_2_-VASc score	2.1 ± 1.6	3.2 ± 1.4	1.3 ± 1.3	<0.001
**Laboratory results**
Creatinine (mg/dl)	1.1 ± 0.4	1.2 ± 0.5	0.9 ± 0.2	<0.001
Glomerular filtration rate (ml/min/1.73 m^2^; CKD-EPI)	76.3 ± 22.1	65.7 ± 21.0	84.5 ± 19.5	<0.001
GOT (U/l)	25.3 ± 11.6	27.2 ± 13.0	23.8 ± 10.3	0.025
GPT (U/l)	32.4 ± 35.6	36.7 ± 50.6	29.2 ± 16.8	0.170
INR	1.1 ± 0.4	1.3 ± 0.6	1 ± 0.3	<0.001
Hemoglobin (g/dl)	13.9 ± 1.5	13.8 ± 1.6	14 ± 1.4	0.414
Leucocytes (Mrd/l)	7.3 ± 2.1	7.4 ± 2.3	7.2 ± 1.9	0.786
Thrombocytes (Mrd/l)	221.7 ± 58.7	204.1 ± 56.6	235 ± 56.9	<0.001
Potassium (mmol/l)	4.1 ± 0.4	4.2 ± 0.4	4.1 ± 0.4	<0.001
**Antiarrhythmic drugs at admission**
Flecainide	4.2%	5.5%	3.3%	0.501
Betablockers	68.1%	83.5%	56.6%	<0.001
Amiodarone	8.5%	18.7%	0.8%	<0.001
**Anticoagulation**
DOAC	9.9%	17.6%	4.1%	0.002
Vit. -K- antagonists	6.1%	9.9%	3.3%	0.079
Platelet inhibitors	23.9%	45.1%	8.2%	<0.001

*Data are presented as per cent or mean ± standard deviation. p < 0.05 is considered statistically significant. Electrical storm was defined as three or more episodes of sustained ventricular arrhythmias in 24 h*.

*CKD-EPI, chronic kidney disease epidemiology collaboration; CRT-D, cardiac resynchronisation therapy defibrillator; DOAC, direct oral anticoagulant; GOT, glutamic-oxaloacetic transaminase; GPT, glutamate pyruvate transaminase; ICD, implanted cardioverter-defibrillator, INR, international normalized ratio; LV-EF, left ventricular ejection fraction; No., number; PVC, premature ventricular complex; TAPSE, tricuspid annular plane systolic excursion; Vit., vitamin*.

### Patients With Outflow Tract Arrhythmias

#### Baseline Parameters

Outflow tract arrhythmias were identified and treated in 215/488 patients (44.1% of the entire cohort, Figure 1). Those patients were of male gender in 64.2% (138/215) and had a mean age of 58.3 ± 16 years. 43.3% (93/215) had SHD, 43.0% (40/93) of those showing ischemic heart disease (Supplementary Table 2). Further baseline parameters of patients with outflow tract arrhythmias are presented in Table 1. Patients with SHD presenting for ablation of outflow tract arrhythmias were older (65.0 ± 12.8 vs. 53.3 ± 16.3 years; *p* < 0.001) und more often of male gender [82.8% (77/93) vs. 50.0% (61/122); *p* < 0.001] than patients without SHD. They were more frequently transferred with urgency to our center [31.2% (29/93) vs. 18.8% (23/122); *p* = 0.036]. Left and right ventricular systolic function was reduced in comparison to patients with outflow tract arrhythmias without SHD (left ventricular ejection fraction: 40 ± 14 vs. 57 ± 8; *p* < 0.001 and TAPSE 20.7 ± 5.9 vs. 23.3 ± 4.8 mm; *p* = 0.001). A higher percentage of patients with SHD also had comorbidities like arterial hypertension, diabetes, and chronic kidney disease (Table 1).

#### Procedural Parameters

Patients with SHD were more likely to be treated for VT than patients without SHD [34.4% (32/93) vs. 18.9% (23/122); *p* =0.012]. Furthermore, ablation in SHD patients was performed more frequently in the left ventricle [64.5% (60/93) vs. 50.0% (61/122); *p* = 0.038; see details of ablation sites in Supplementary Table 3]. Procedure duration [142 (84) vs. 105 (72) min; *p* < 0.001] as well as fluoroscopy and energy delivery time were longer in patients with SHD (Table 2). The number of inducible VT or PVC targeted differed only slightly between groups. Three patients (two with SHD) had an epicardial ablation.

**Table 2:** Procedural parameters of patients with outflow tract arrhythmias.

**Variable**	**All** **(*n* = 215)**	**With structural heart disease** **(*n* = 93)**	**Without structural heart disease** **(*n* = 122)**	***p*** **-value**
**Clinical target**
PVC	83.3%	72.0%	91.8%	<0.001
VT	25.6%	34.4%	18.9%	0.012
**Outflow tract origin**
RVOT	49.3%	43.0%	54.1%	0.130
LVOT	60.0%	68.8%	53.3%	0.025
Procedure duration (minutes)	132.3 ± 62.1 122.5 (91)	150.5 ± 61.7 142 (84)	118.7 ± 59.1 105 (72)	<0.001
Fluoroscopy duration (seconds)	760.1 ± 559.2 641.5 (690.5)	963.1 ± 617.0 826 (730)	608.3 ± 458.7 429 (562)	<0.001
Fluoroscopy dose (cGycm^2^)	605 ± 1,309 279 (492)	952 ± 1,873 568 (770)	343 ± 461 186 (288)	<0.001
Radiofrequency energy duration (seconds)	661.4 ± 559.7 465 (682)	833.3 ± 643.6 621 (974)	534.7 ± 451.2 360.5 (407)	<0.001
Contrast dye (milliliters)	10.4 ± 18.0	12.6 ± 18.4	8.8 ± 17.6	0.087
No. of VTs induced	1.9 ± 1.5	2.0 ± 1.5	1.6 ± 1.4	0.140
No. of targeted PVCs	1.5 ± 1.1	1.7 ± 1.2	1.4 ± 1.1	0.033
**Previous ablation for ventricular arrhythmia**	22.3%	32.3%	14.8%	0.003
Thereof of same target(s)	42.6%	51.7%	27.8%	0.137
General anesthesia	4.2%	6.5%	2.5%	0.180
Catecholamines during procedure	4.2%	7.6%	1.6%	0.041
ECMO/Impella during procedure	0.9%	2.2%	0%	0.186
Epicardial approach	1.4%	2.2%	0.8%	0.580
Ablation in RV	55.8%	50.5%	59.8%	0.212
Ablation in LV	56.3%	64.5%	50.0%	0.038
Ablation in CS	13.5%	17.2%	10.7%	0.226

*Data are presented as per cent, mean ± standard deviation or median (IQR). p < 0.05 is considered statistically significant*.

*CS, coronary sinus; ECMO, extracorporeal membrane oxygenation; LV, left ventricle; LVOT, left ventricular outflow tract; No., number; PVC, premature ventricular complex; RV, right ventricle; RVOT, right ventricular outflow tract; VT, ventricular tachycardia*.

### Procedural Outcome of Patients With and Without Structural Heart Disease

The acute success rate was high in both groups with 93.4% (86/93) vs. 94.2% (115/122) (SHD vs. no SHD; *p* = 0.781). Early recurrences of the targeted arrhythmia were observed in 16.1% (15/93) vs. 9.0% (11/122; *p* = 0.140) and there was a numerically higher rate of reablations during the stay in patients with SHD [4.3% (4/93) vs. 0.8% (1/122); *p* = 0.168].

Periprocedural complications occurred in 12.9% (12/93) vs. 6.6% (8/122) of patients (SHD vs. no SHD; *p* = 0.154). Complications of the groin were seen more often in patients with SHD [9.7% (9/93) vs. 4.1% (5/122); for details see Supplementary Table 4]. There was no significant association between a retrograde aortic mapping approach and groin complications: arterial groin puncture was performed in 60.7% (128/211) of all patients and 66.7% (10/15) of patients with groin complications (*p* = 0.787). A pericardial tamponade that required drainage with a pigtail catheter was observed in one patient without SHD (0.8%). A full list of all complications is provided in Supplementary Table 4. One patient with cardiac sarcoidosis died during the post-interventional stay due to progressive heart failure (Table 3).

**Table 3:** Acute outcome of patients with outflow tract arrhythmias.

**Variable**	**All** **(*n* = 215)**	**With structural heart disease** **(*n* = 93)**	**Without structural heart disease** **(*n* = 122)**	***p*** **-value**
Acute success	94.0%	93.4%	94.2%	0.781
**Periprocedural complication**	9.3%	12.9%	6.6%	0.154
Groin complications	6.5%	9.7%	4.1%	
Pericardial tamponade	0.5%	0%	0.8%	
Periprocedural death	0.5%	1.1%	0.0%	0.433
Reablation during stay	2.3%	4.3%	0.8%	0.168
Recurrence during stay	12.1%	16.1%	9.0%	0.140
Discharge to other hospital	1.4%	2.3%	0.8%	0.572
Length of stay (total; nights)	4.9 ± 5.7 2 (4)	6.5 ± 7.1 5 (6)	3.8 ± 4.1 2 (4)	<0.001
Postinterventional acute heart failure	1.4%	3.3%	0.0%	0.077
Postinterventional acute kidney injury	3.8%	7.7%	0.8%	0.022
**Antiarrhythmic drugs at discharge**
Flecainide	3.4%	1.1%	5.0%	0.133
Betablockers	67.8%	85.1%	55.4%	<0.001
Amiodarone	6.7%	16.1%	0.0%	<0.001

*Data are presented as per cent, mean ± standard deviation or median (IQR). p < 0.05 is considered statistically significant*.

The median duration of stay was 5 (6) nights for patients with SHD and 2 (4) nights for otherwise healthy patients (*p* < 0.001). Changes in antiarrhythmic medication at discharge were insignificant in both groups compared to the initial admission (see Supplementary Figure 1).

**Figure 2:**
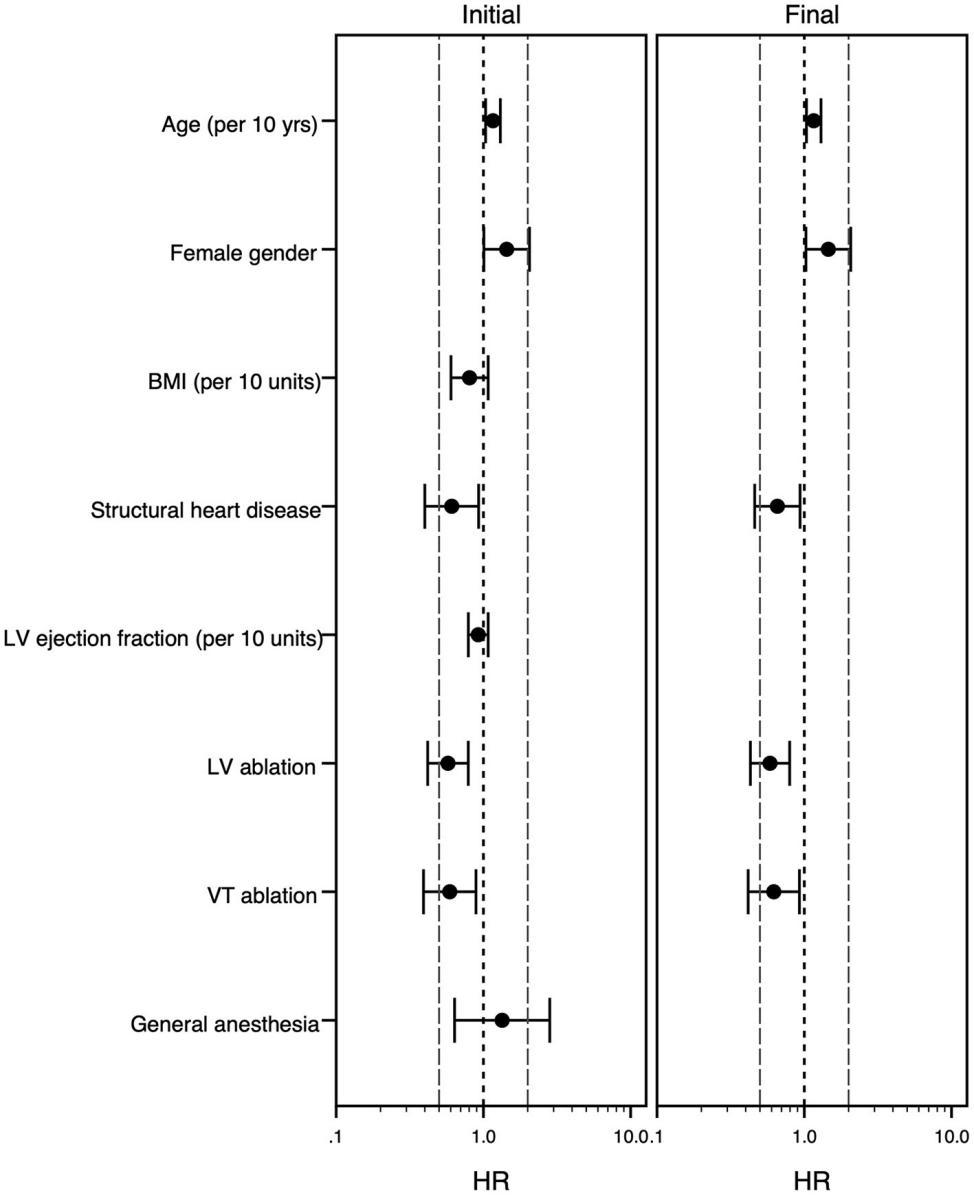
Forest plot of the multivariable Cox model analyzing risk factors for longer procedure duration. The multivariable Cox-regression analysis is displayed. Hazard ratio <1 stands for association with longer procedure duration, while hazard ratio >1 goes along with shorter procedure duration. The initial model (left panel) and final model (right panel) is shown. The grouping variable “structural heart disease” was forced into the model equations, all other covariates were selected following the forward-stepwise variable selection method. For age, body mass index and left ventricular ejection fraction the effect of an increase of 10 years/kg/m^2^/% is shown. Hazard ratios (point) with 95% confidence intervals (whiskers) are displayed. BMI, body mass index (kg/m^2^); HR, hazard ratio; LV, left ventricle; VT, ventricular tachycardia, yrs., years.

**Figure 3:**
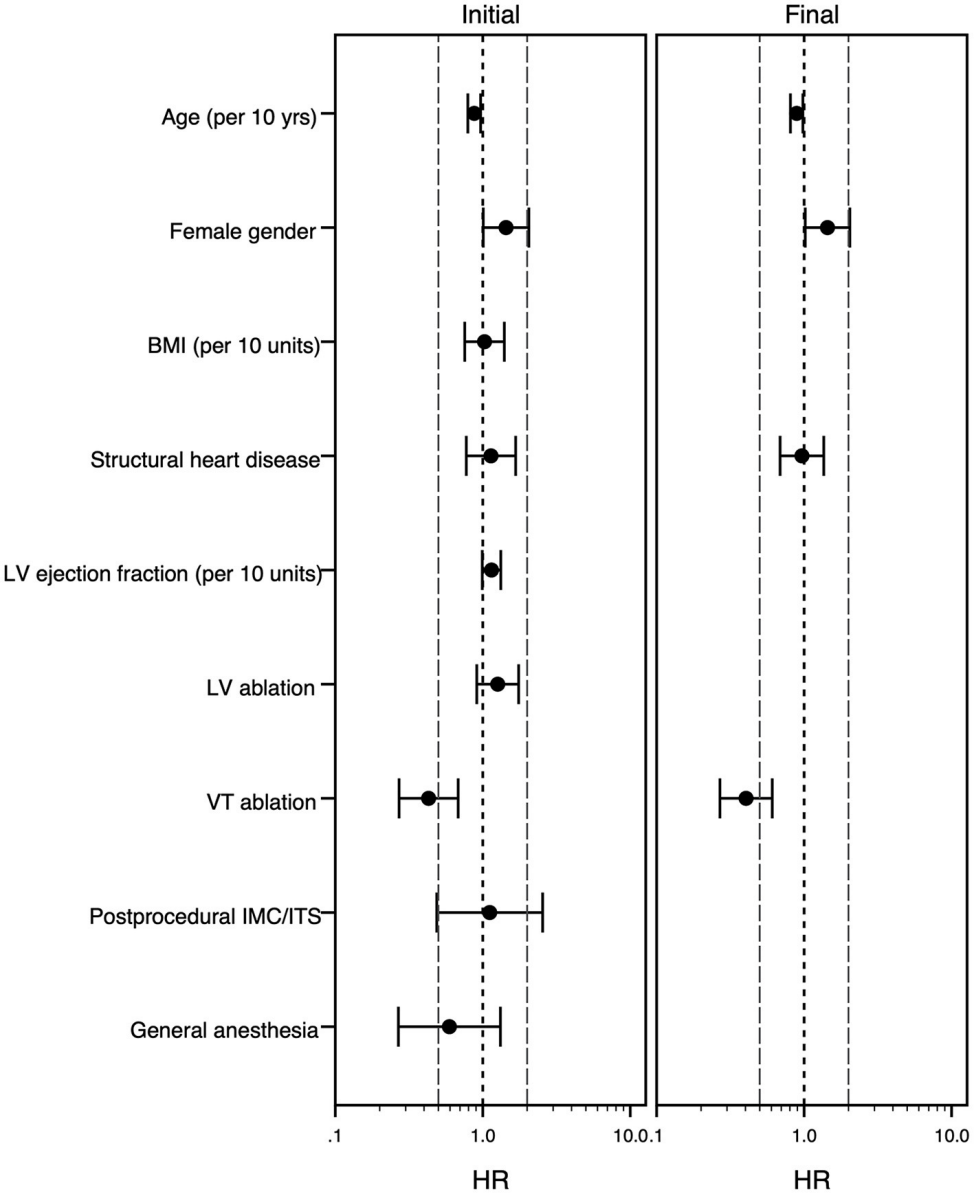
Forest plot of the multivariable Cox model analyzing risk factors for longer hospital stay. The multivariable Cox-regression analysis is displayed. Hazard ratio <1 stands for association with longer hospital stay, while hazard ratio >1 goes along with shorter stay. The initial model (left panel) and final model (right panel) is shown. The grouping variable “structural heart disease” was forced into the model equations, all other covariates were selected following the forward-stepwise variable selection method. For age, body mass index and left ventricular ejection fraction the effect of an increase of 10 years/kg/m^2^/% is shown. Hazard ratios (point) with 95% confidence intervals (whiskers) are displayed. BMI, body mass index (kg/m^2^); HR, hazard ratio; IMC/ITS, intermediate care unit/intensive care unit; LV indicates left ventricle; VT, ventricular tachycardia, yrs., years.

In the univariate analysis, SHD was associated with a longer procedure duration as well as a longer hospital stay and there was a trend toward higher likelihood of periprocedural complications (Table 4A). The multivariable analysis confirmed this result regarding procedure duration, but not regarding length of stay. Additionally, ablation of VT instead of PVC and ablation within the left ventricle were identified as factors independently associated with a longer procedure duration, while female gender and higher age were associated with a shorter procedure duration. Regarding the length of stay, higher age and VT ablation were associated with a longer stay, while female gender was associated with a shorter stay at the hospital (Table 4B; Figures 2, 3).

**Table 4:** Uni- and multivariable analysis.

**Variable**	**Influencing factors**	**Hazard ratio**	**Confidence interval**	***p*** **-value**
**(A) Univariate analysis**				
**Procedural duration**
	Structural heart disease	0.594	0.439–0.804	<0.001
**Length of hospital stay**
	Structural heart disease	0.696	0.517–0.936	0.017
**Periprocedural complications**
	Structural heart disease	2.107	0.927–4.796	0.076
**(B) Multivariable analysis**				
**Procedure duration**
	Age	1.015	1.003–1.027	0.001
	Female gender	1.457	1.027–2.069	0.035
	Structural heart disease	0.656	0.461–0.936	0.02
	VT ablation	0.621	0.416–0.926	0.02
	LV ablation	0.585	0.431–0.795	<0.001
**Length of hospital stay**
	Age	0.988	0.979–0.998	0.018
	Female gender	1.441	1.017–2.042	0.040
	Structural heart disease	0.966	0.687–1.359	0.844
	VT ablation	0.404	0.268–0.608	<0.001

*Uni- (**A**) as well as multivariable (**B**) Cox-regression analyses were performed. Lower hazard ratio stands for association with longer procedure duration as well as longer hospital stay, while higher hazard ratio goes along with shorter procedure duration, length of stay and a higher likelihood of periprocedural complications. In the multivariable analyses, the grouping variable “structural heart disease” was forced into the model equations while all other covariates were selected following the forward-stepwise variable selection method. The initial model for procedure duration consisted of age, body-mass-index, left ventricular ejection fraction, female gender, structural heart disease, VT ablation, procedure under general anesthesia and LV ablation. The initial model for length of hospital stay consisted of age, body-mass-index, left ventricular ejection fraction, gender, structural heart disease, VT ablation, procedure under general anesthesia, LV ablation and postoperative intermediate or intensive care treatment. Hazard ratios with 95% confidence intervals as well as Wald p-values are displayed. P-values < 0.05 are regarded statistically significant*.

*LV, left ventricle; VT, ventricular tachycardia*.

## Discussion

The main findings of the present study are:

- Outflow tract arrhythmias are common in patients with SHD. Those patients presenting for ablation are older, more often male and have more comorbidities than patients with idiopathic ventricular arrhythmias.
- The acute procedural success of outflow tract arrhythmia ablation is similar in patients with and without SHD.
- Outflow tract arrhythmias in patients with SHD have their origin more often in the left ventricle than outflow tract arrhythmias in patients without SHD.
- The median duration of hospital stay was 3 days longer in patients with SHD than in patients without SHD. Patients with SHD had numerically more periprocedural complications.

The present study shows that almost half of the patients being treated for outflow tract arrhythmias also suffer from underlying SHD. The evidence available on outflow tract arrhythmias in patients with SHD is still limited, as those patients have so far often been excluded from most clinical trials. Our study is the first, to compare the procedural parameters and acute outcome of outflow tract arrhythmias in patients with and without SHD. A proper prediction of those parameters seems to be especially relevant in times with restricted availability of hospital capacities due to the COVID-19 pandemic.

Two previous publications investigated the origin and outcome of ventricular arrhythmias unrelated to myocardial substrate in patients with SHD, showing, that “idiopathic arrhythmias” remote from abnormal myocardial substrate do occur in those patients and have an acceptable ablation outcome (17, 18). Arrhythmias associated with abnormal myocardial substrate on the other hand tend to have a worse ablation outcome, but several other confounding factors such as the accessibility of area of origin of the arrhythmia might be important (4). The procedural parameters and ablation outcome of outflow tract arrhythmias, representing a typical location of idiopathic arrhythmias, but also an area that might be affected by abnormal myocardial substrate remains unclear. This is especially relevant for patients with non-ischemic cardiomyopathy, as areas of fibrosis can sometimes be found associated to the outflow tracts in those individuals during magnetic resonance imaging (11, 19, 20).

According to our analysis, patients with SHD presenting with outflow tract arrhythmias differ in most baseline parameters from patients without SHD. In brief, they are older, more often male and have higher counts of comorbidities like arterial hypertension and diabetes. Factors, recently proven to be relevant indicators of 1-year mortality and recurrence like left ventricular function, prior ICD implantation and previous ablation (I-VT score) were observed more often in comparison to patients without SHD (21). In our cohort, rates of complications and intrahospital death were comparable to previously published large trials. The higher amount of groin complications in the SHD group might be attributed to a slightly higher percentage of arterial punctures in this group in combination with more patients on oral anticoagulation for treatment of atrial fibrillation. Furthermore, a higher body-mass-index might play a role, even if experiences from catheter ablation of atrial fibrillation only showed an effect for the morbidly obese (22).

Procedures in the SHD group took on average 32 min longer than procedures in otherwise healthy patients. This can potentially be explained by differences during the sedation as well as the ablation procedure itself. Furthermore, there was a higher amount of VT and arrhythmias with origin in the left ventricle and in these patients an arterial access and invasive blood pressure monitoring was used. At last, in patients with SHD there was a trend to more VT morphologies and PVCs to be targeted during the procedure. The latter might be caused by the myocardial substrate itself being a source of a variety of arrhythmias, while the mechanism of arrhythmia in otherwise healthy patients was most likely automaticity or triggered activity (23).

The acute success rate was equally high in both groups, which fits with results from previous studies that show good acute success rates for patients with SHD, even if the long-term results were less optimal (4). Early recurrences occurred numerically more often in patients with SHD without reaching statistical significance.

The overall length of the hospital stay was 5 nights (median) for patients with SHD and thus longer than in patients without SHD. Recently published data on ablation of non-ischemic VT shows a similar duration of stay (24). The difference between groups might be caused by comorbidities (7.7% of acute kidney failure and 3.3% cardiac decompensation) in the SHD group, but also by a longer surveillance after femoral artery puncture. The multivariable analysis suggests that higher age and ablation of VT are independent factors associated with a longer hospital stay. However, those factors also seem to be associated with a generally sicker patient collective which requests a more extent periprocedural surveillance period. Female gender was interestingly associated with shorter procedure duration as well as hospital stay, potentially reflecting, that female patients were younger and had less comorbidities than male patients.

## Limitations

The data for this analysis has been derived from a single center registry and was analyzed retrospectively. Our results should therefore be considered as hypothesis generating and might not be applicable to the general population.

## Conclusion

This analysis shows that patients presenting with outflow tract arrhythmias and coexisting SHD have a higher burden of risk factors and comorbidities that might be relevant for ablation procedures than patients without SHD. The acute success rate was high, irrespective of the presence of SHD, but a longer hospital stay and potentially a higher risk of periprocedural complications should be considered when discussing this treatment option with patients.

## Data Availability Statement

The raw data supporting the conclusions of this article will be made available by the authors, upon reasonable request.

## Ethics Statement

The studies involving human participants were reviewed and approved by Ethics Committee of the Medical Association Hamburg. The patients/participants provided their written informed consent to participate in this study.

## Author Contributions

RS and JR were involved in data generation, data collection, study design, statistical analysis, and manuscript preparation. AB was involved in data generation, data collection, and manuscript preparation. HP was involved in study design, statistical analysis, and manuscript preparation. LR, FM, JM, SK, IM, ML, BR, CM, AM, FO, PK, and AR were involved in data generation, data analysis, and manuscript preparation. All authors contributed to the article and approved the submitted version.

## Conflict of Interest

The authors declare that the research was conducted in the absence of any commercial or financial relationships that could be construed as a potential conflict of interest.

## Publisher's Note

All claims expressed in this article are solely those of the authors and do not necessarily represent those of their affiliated organizations, or those of the publisher, the editors and the reviewers. Any product that may be evaluated in this article, or claim that may be made by its manufacturer, is not guaranteed or endorsed by the publisher.
