# Supplementary material for: Ablation of Outflow Tract Arrhythmias in Patients With and Without Structural Heart Disease—A Comparative Analysis
Source: Front Cardiovasc Med. 2022 May 25;9:910042. doi: 10.3389/fcvm.2022.910042 (PMC9174508; doi:10.3389/fcvm.2022.910042)
Supplement: Supplementary file 1 [file Data_Sheet_1.docx]

**Supplement**

**Supplementary Table 1: Baseline characteristics of all patients**

| **Variable** | **All (n=488)** | **With structural heart disease (n=306)** | **Without structural heart disease (n=182)** |
| --- | --- | --- | --- |
| Age (years) | 61.1±15 | 65.4±12.1 | 54±16.5 |
| Body-mass-index (kg/m^2^) | 27.8±5.1 | 28.7±5.1 | 26.4±4.8 |
| Gender (male) | 71.7% | 82.2% | 53.8% |
| Structural heart disease | 62.7% | 100% | 0.0% |
| Ischemic |  | 62.7% |  |
| Non-ischemic |  | 37.2% |  |
| LV-EF (%) | 44±15 | 37±13 | 56±9 |
| TAPSE (mm) | 20.7±5.3 | 19.4±5.1 | 23.2±4.6 |
| Arterial hypertension | 52.0% | 62.7% | 34.1% |
| Diabetes mellitus | 15.8% | 20.6% | 7.7% |
| Chronic kidney disease | 36.7% | 48% | 17.6% |
| Chronic lung disease | 18.4% | 23.2% | 10.4% |
| Chronic liver disease | 5.1% | 6.5% | 2.7% |
| ICD | 41.2% | 59.5% | 10.4% |
| Creatinine (mg/dl) | 1.2±0.5 | 1.3±0.6 | 0.9±0.2 |
| Glomerular filtration rate (ml/min/1.73m^2^) | 69.8±23.7 | 61.8±21.4 | 83.3±21.2 |
| GOT (U/l) | 35.5±108.7 | 42.2±136.8 | 24.3±9.9 |
| GPT (U/l) | 38.4±86.9 | 43.5±108.8 | 30.0±19.2 |
| INR | 1.2±0.5 | 1.3±0.6 | 1.1±0.3 |
| Haemoglobin (g/dl) | 13.7±1.6 | 13.5±1.7 | 14.0±1.4 |
| Leucocytes (Mrd/l) | 7.7±2.4 | 7.9±2.6 | 7.3±1.9 |
| Thrombocytes (Mrd/l) | 219.0±65.3 | 210.3±67.1 | 233.5±59.4 |
| Potassium (mmol/l) | 4.2±0.5 | 4.3±0.5 | 4.1±0.4 |
| Antiarrhythmic drugs |  |  |  |
| Flecainide | 2.9% | 2.6% | 3.3% |
| Betablockers | 74.9% | 85.9% | 56.6% |
| Amiodarone | 17.5% | 27.0% | 1.6% |
| Anticoagulation |  |  |  |
| DOAC | 15.0% | 19.7% | 7.1% |
| Vit. K antagonists | 9.3% | 12.5% | 3.8% |
| Platelet inhibitors | 32.9% | 48.0% | 7.7% |

Data are presented as per cent or mean ±standard deviation.

CPR indicates cardiopulmonary resuscitation; DOAC, direct oral anticoagulant; GOT, glutamic-oxaloacetic transaminase; GPT, glutamatate pyruvate transaminase; ICD, implanted cardioverter-defibrillator, INR, international normalised ratio; LV-EF, left ventricular ejection fraction; TAPSE, tricuspid annular plane systolic excursion; Vit., vitamin.

**Supplementary Table 2: Types of cardiomyopathy in patients with structural heart disease**

| **Type of Cardiomyopathy** | **Percentage of patients with structural heart disease** |
| --- | --- |
| Ischemic cardiomyopathy | 43.0% |
| Non-ischemic cardiomyopathy | 57.0% |
| Dilative cardiomyopathy | 43.2% |
| Hypertrophic cardiomyopathy | 1.1% |
| Cardiac sarcoidosis | 2.2% |
| Other | 6.5% |

Data are presented as per cent.

**Supplementary Table 3: List of catheter ablation target sites**

| **Ablation site** | **Percent of all patients (n=215)** |
| --- | --- |
| Left ventricle |  |
| LVOT | 10.7% |
| Basal septal LV | 13.0% |
| Great cardiac vein | 6.0% |
| LV-summit | 11.6% |
| Aortomitral continuity | 3.3% |
| LCC | 12.1% |
| RCC | 5.6% |
| NCC | 2.3% |
| Right ventricle |  |
| RVOT | 50.7% |

Ablation sides are displayed. Values are presented as per cent.

LCC indicates left coronary cusp; LV, left ventricle; LVOT, left ventricular outflow tract, NCC, noncoronary cusp; RCC, right coronary cusp; RV, right ventricle; RVOT, right ventricular outflow tract.

**Supplementary Table 4: List of periprocedural complications of patients with outflow tract arrhythmias**

| **Variable** | **All (n=215)** | **With structural heart disease (n=93)** | **Without structural heart disease (n=122)** |
| --- | --- | --- | --- |
| Groin complication | 6.5% | 9.7% | 4.1% |
| Thereof with interventional treatment | 64.3% | 77.8% | 40.0% |
| Aneurysma spurium | 3.7% | 5.4% | 2.5% |
| AV-fistula | 1.4% | 2.2% | 0.8% |
| Hypoesthesia | 0.5% | 1.1% | 0.0% |
| Dislocation of closure device | 0.5% | 1.1% | 0.0% |
| Vein thrombosis | 0.5% | 0.0% | 0.8% |
| Pericardial effusion | 0.5% | 0% | 0.8% |
| Stroke | 0.5% | 1.1% | 0.0% |
| Infections (aspiration pneumonia, UTI) | 1.9% | 3.2% | 0.8% |
| Severe heart failure | 0.5% | 1.1% | 0.0% |
| Total AV-block | 0.5% | 1.1% | 0.0% |
| Severe kidney failure | 0.5% | 1.1% | 0.0% |
| Urinary retention | 0.5% | 1.1% | 0.0% |
| Cardiopulmonary resuscitation | 0.5% | 1.1% | 0.0% |
| Pulmonary embolism | 0.5% | 0.0% | 0.8% |

Data are presented as per cent.

AV indicates atrio ventricular; UTI, urinary tract infection.

**Supplementary Figure 1: Comparison of antiarrhythmic drug therapy at admission and at discharge**

**A Patients with structural heart disease**

**
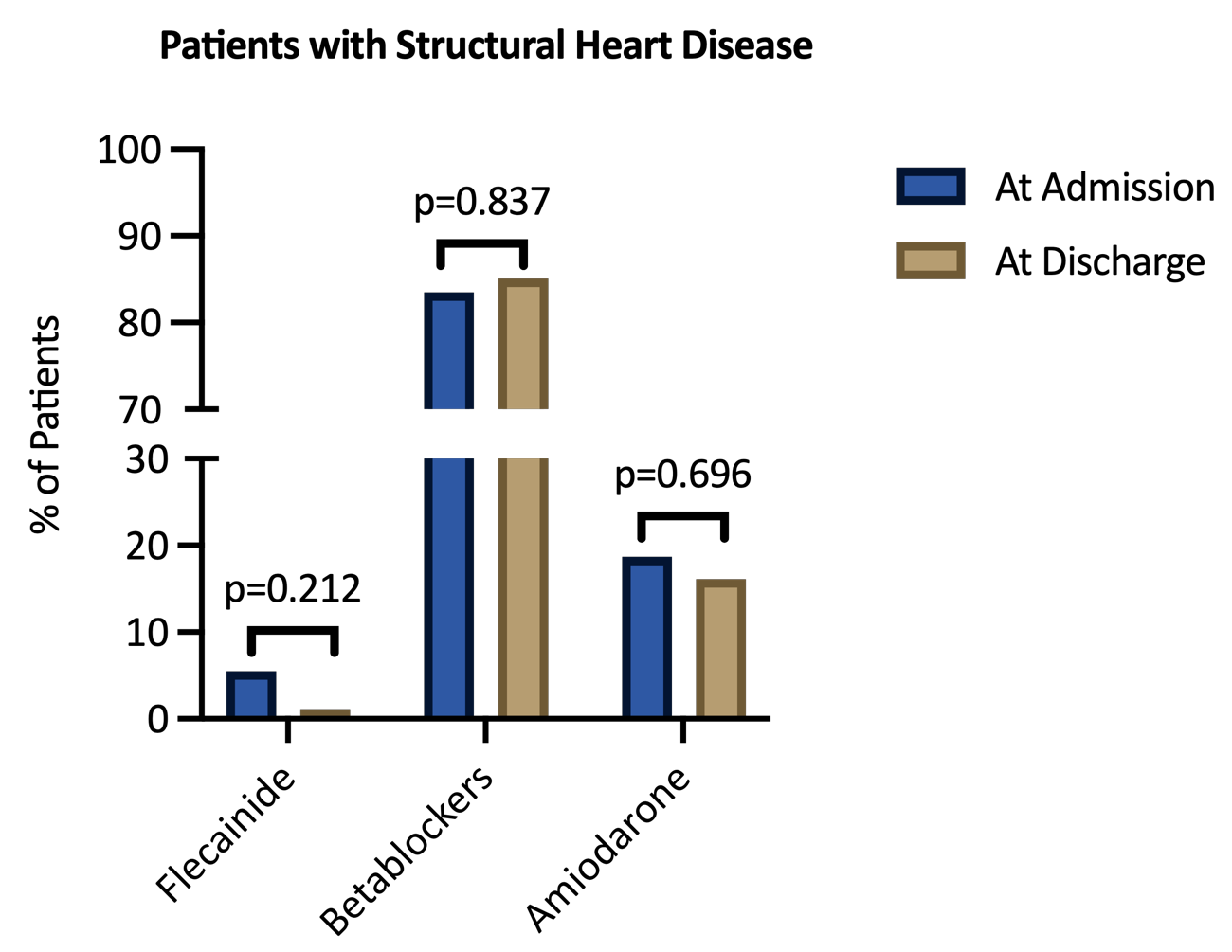
**

**B Patients without structural heart disease**

**
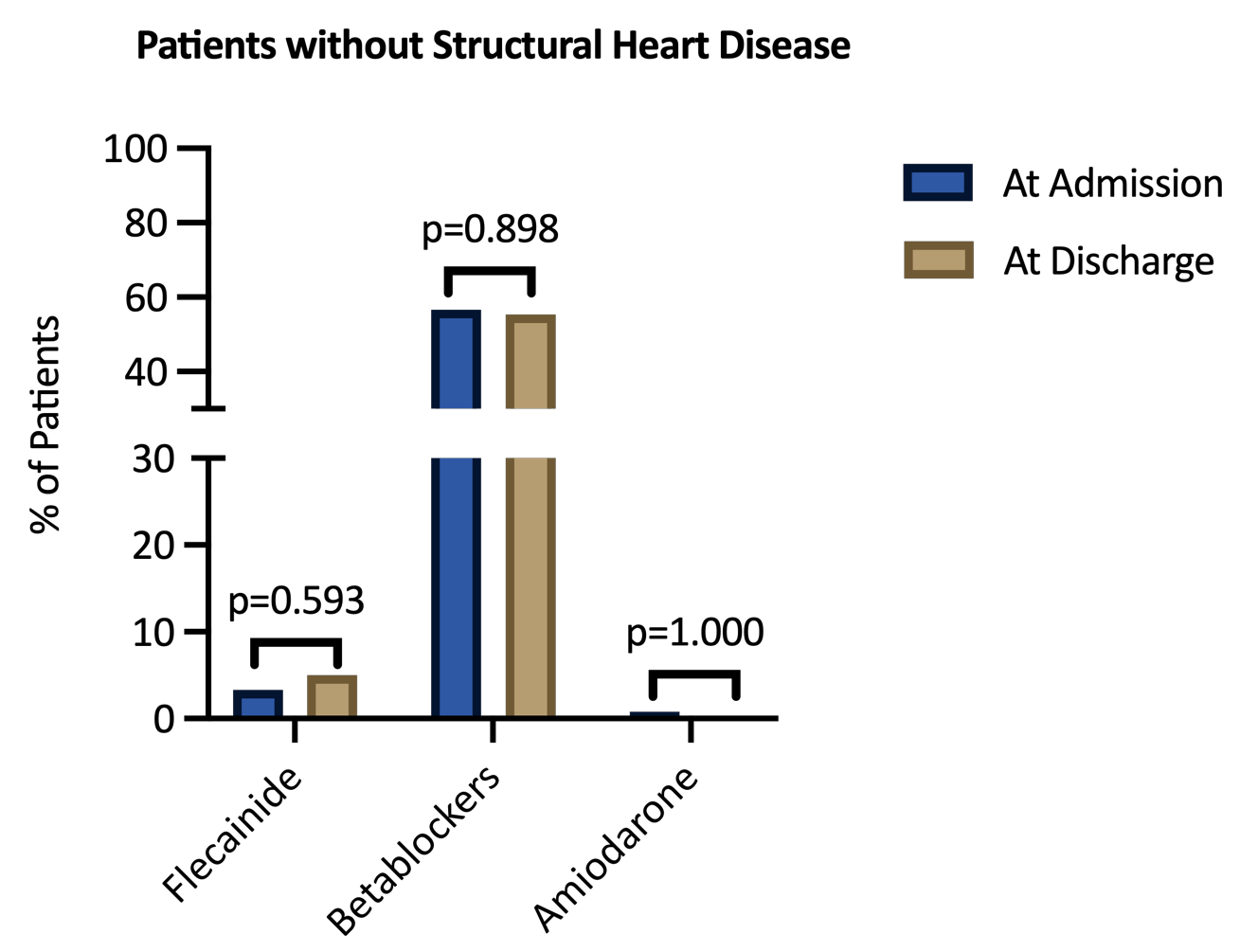
**

The rate of antiarrhythmic drug therapy before (blue bars) and after (brown bars) the procedure in patients with (**A**) and without (**B**) structural heart disease was compared. There were no significant differences. p <0.05 is considered statistically significant.
